# Deep Margin Elevation: A Literature Review

**DOI:** 10.3390/dj10030048

**Published:** 2022-03-14

**Authors:** Theodora Kalliopi Samartzi, Dimokritos Papalexopoulos, Panagiotis Ntovas, Christos Rahiotis, Markus B. Blatz

**Affiliations:** 1 Private Practice, 115 27 Athens, Greece; 2 Department of Prosthodontics, National and Kapodistrian University of Athens, 115 27 Athens, Greece; dimokpapalex@dent.uoa.gr; 3 Department of Operative Dentistry, National and Kapodistrian University of Athens, 115 27 Athens, Greece; pan.ntovas@dent.uoa.gr (P.N.); craxioti@dent.uoa.gr (C.R.); 4 Department of Preventive and Restorative Sciences, University of Pennsylvania School of Dental Medicine, 240 S 40th St., Philadelphia, PA 19104, USA; mblatz@upenn.edu

**Keywords:** deep margin elevation, proximal box elevation, cervical margin relocation, dental caries, subgingival margins

## Abstract

A conservative approach for restoring deep proximal lesions is to apply an increment of composite resin over the preexisting cervical margin to relocate it coronally, the so-called “deep margin elevation” (DME). A literature search for research articles referring to DME published from January 1998 until November 2021 was conducted using MEDLINE (PubMed), Ovid, Scopus, Cochrane Library and Semantic Scholar databases applying preset inclusion and exclusion criteria. Elevation material and adhesive system employed for luting seem to be significant factors concerning the marginal adaptation of the restoration. This technique does not affect bond strength, fatigue behavior, fracture resistance, failure pattern or repairability. DME and subgingival restorations are compatible with periodontal health, given that they are well-polished and refined. The available literature is limited mainly to in vitro studies. Therefore, randomized clinical trials with extended follow-up periods are necessary to clarify all aspects of the technique and ascertain its validity in clinical practice. For the time being, DME should be applied with caution respecting three criteria: capability of field isolation, the perfect seal of the cervical margin provided by the matrix, and no invasion of the connective compartment of biological width.

## 1. Introduction

The dental clinician has consistently challenged the restoration of deep proximal lesions since they are usually associated with significant defects with subgingival margins exceeding cementoenamel junction (CEJ) [1,2]. In this clinical scenario, indirect restorations are preferable since they provide better esthetic, anatomic form, physical and mechanical properties, and reduced polymerization shrinkage due to their extraoral fabrication that permits the relief of residual stresses [3,4,5,6,7]. However, subgingival margins remain a challenge as they are challenging to handle due to limited access, rubber dam slippage over the margin, and subsequent persistent saliva, crevice fluid and blood leakage [8].

The conventional approach includes orthodontic extrusion, surgical exposure of the cervical margin, or a combination of both techniques leading to an apical displacement of supporting tissues to access the subgingival margin and obtain adequate space for the establishment of biological width (BW) [1,9,10,11]. Frequently, the techniques mentioned above may cause further attachment loss and exposure of root concavities and furcations to the oral environment, dentin hypersensitivity, and unfavorable crown to root ratio as well as compromised esthetics. Additionally, this process may often delay the delivery of the final restoration [1,9,10,11].

An alternative and more conservative approach, the so-called “deep margin elevation” (DME), is to apply a base of composite resin over the preexisting cervical margin to relocate it coronally [8,12]. This technique introduced in 1998 by Dietschi and Spreafico [12] is also referred to with the terms “cervical margin relocation”, “proximal box elevation”, and “coronal margin relocation”, and presents several benefits concerning proper isolation with a rubber dam and subsequent moisture control, facilitation in impression taking, proper bonding procedures, and excess removal and avoidance of unnecessary tissue sacrifice [2,8,13]. The open-sandwich technique is widely considered the forerunner of DME [14]—introduced to overcome sealing issues in deep Class II direct composite restorations, the open-sandwich technique uses a glass ionomer or resin-modified glass ionomer to fill the cervical part of the box, which results in a part of the glass ionomer/resin-modified glass ionomer being exposed to the oral environment [14,15]. However, even if the two techniques resemble each other, DME was initially described for indirect restorations using composite resin [12]. Today, DME can be combined with immediate dentin sealing (IDS) to improve indirect adhesive restorations’ bond and marginal seal. The adhesive composite resin base is used to seal the dentin, correct geometry, reinforce undermined cusps, and fill undercuts.

Even if DME seems a valuable technique, clinicians have not extensively applied it. The reluctancy around DME could be attributed to the insufficient available literature to provide clear answers regarding the topic; most studies focus on specific aspects of DME like technique presentation and marginal adaptation, while recent articles elaborating all of the existing knowledge are lacking. Therefore, this study aims to review the literature and clarify whether DME is a reliable technique to adopt in clinical practice.

## 2. Materials and Methods

A search in the literature was conducted for evidence-based research articles referring to DME published from January 1998 until November 2021 using MEDLINE (PubMed), Ovid, Scopus, Cochrane Library and Semantic Scholar databases. Furthermore, the reporting scheme of this review aligned to the recommendations of those of the PICO framework. The following terms were used as key words: “deep margin elevation”, “proximal box elevation”, “cervical margin relocation”, and “coronal margin relocation”. Supplementary manual research was also performed, screening the references from the articles that emerged from the initial selection.

The eligibility criteria were:

Study design: clinical (in vivo), in vitro studies, case studies, and reviews referring to the DME technique.
Type of teeth: no restriction. Studies referring to human permanent teeth were included.
Target condition: any study investigating DME.
Inclusion criteria: only studies reporting on sensitivity and specificity values were included.
Language: Peer reviewed papers written in English

After a gradual screening of titles, abstracts, and full texts, two reviewers evaluated all articles independently for their appropriateness (T.K.S. and D.P.). Discrepancies between the two reviewers were discussed until a consensus was reached.

## 3. Results

**Figure 1:**
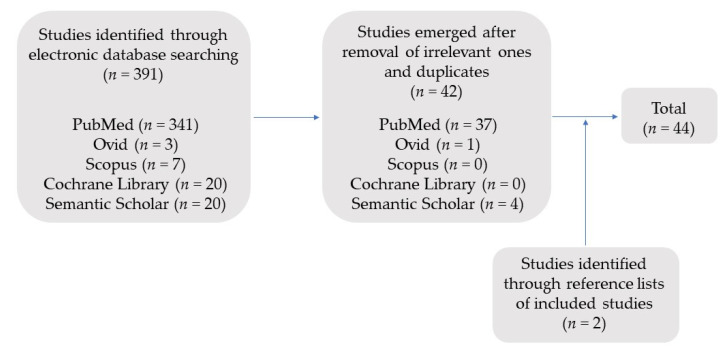
Process of final studies selection.

The initial search identified 391 articles. Ultimately, 44 articles were included in the present review, after excluding irrelevant ones or duplicates (Figure 1). Information about the authors/year of publication, the type of study, the study design, and the main findings are summarized and depicted in Table 1. Most of the studies focused on technique description, the microleakage/marginal adaptation, and the mechanical performance of the final restoration after the application of DME and its compatibility with periodontal tissues. For better understanding, we segmented literature accordingly.

### 3.1. DME Protocol

The initial step to restoring deep proximal cavities is to assess the extent of the carious lesion or the extension of the crack, its proximity to the pulp and the distance of the future therapeutic margin from the bone crest. For this purpose, measurement of probing depth, bone sounding and periapical radiographs are required preoperatively [8,16].

DME can be performed in all cases of deep proximal lesions when the following criteria are satisfied; first, the working field should be completely isolated. Second, the matrix should isolate margins accurately and ensure a perfect seal around them. Third, the connective compartment of BW must not be violated by the matrix [8,24].

When conditions are met, the carious defect is removed, and a circumferential stainless-steel matrix is applied around the tooth to seal the cervical margin [18,21]. Curved matrices are preferable since they provide a better gingival emergence profile compared to traditional ones [17]. The presence of sufficient tooth substance at both buccal and lingual walls is a prerequisite for the stability of the matrix; instability is equal to failure of the technique and, in that case, the treatment plan needs to be reconsidered [8]. The matrix dimensions should be higher than the desired elevation level but narrow enough to slip easily in the subgingival area. For this reason, it might need to be reduced 2–3 mm with scissors [8]. In the case of severely deep localized lesions, the “matrix-in-a matrix” technique seems beneficial: a sectional matrix is inserted vertically into the subgingival area through a loosened Tofflemire or Apis matrix; when reaching the deepest level of the defect, the Tofflemire or Apis matrix is secured [8]. Then, a wedge with an adequate 3D anatomy is inserted. If the wedge affects the profile of the matrix, Teflon can be packed instead [52].

No rubber dam or gingival tissue should interfere between the cavity margin and the matrix [8]. The margin is carefully re-prepared with oscillating diamond tips or fine diamond burs [56].

Afterwards, a thick layer of a dentin bonding agent (DBA) is applied on the exposed dentin and light-polymerized according to manufacturer instructions (immediate dentin sealing-IDS) [57]. A supplementary layer of low-viscosity resin is imperative in the case of unfilled DBAs [57,58]. Then, the deep margin is elevated using flowable or condensable composite or a combination of both [12,17,19]. In the case of micro-hybrid or nano-hybrid composites, preheating is suggested to eliminate interlayer gaps and further facilitate placement [8]. The amount of composite should be the minimum needed for the elevation [19]. Composite can also be used to correct geometry and eliminate undercuts (Cavity design Optimization) [12,17,19,27]. Final polymerization through glycerin gel is strongly recommended to eliminate oxygen inhibition layer (OIL) (air-blocking).

Subsequently, the preparation is rinsed with air–water spray, the enamel margins are re-prepared, and the composite excesses are gently removed and polished (with a sickle or a No. 12 blade). A postoperative bitewing radiograph is of utmost importance to ensure the absence of gaps or overhangs and to proceed to the final preparation and impression taking (conventional or optical impressions) [8]. At the cementation appointment, the existing composite and IDS surface need to be cleaned and air-abraded and the restoration is cemented according to manufacturer instructions [59].

Given that subgingival caries that exceed CEJ constitute significant defects and are usually accompanied by severe coronal destruction, direct restorations are contraindicated. However, even if DME was initially performed with indirect restorations, in case of localized deep lesions or when a patient cannot afford an indirect restoration, a direct approach should be reconsidered [16,24]. Therefore, DME would be the preliminary stage for an extensive composite restoration facilitating the rubber dam placement and the adjustment of separation rings, thus achieving tight contacts and satisfying proximal contours [16,24]. Furthermore, if severely damaged teeth are missing three or more surfaces, DME combined with IDS and delayed composite placement is preferable instead of completing it at the same appointment [8].

### 3.2. Marginal Adaptation/Microleakage

The ideal substrate for bonding of an adhesive restoration is enamel [60,61,62,63]. In the subgingival area, enamel diminishes gradually and, beyond CEJ, the cavity margin consists of dentin and cementum that deteriorate bonding quality [2,60,61,62,63].

Microleakage constitutes a significant factor that determines restoration success [64] mainly when restorative margins are located apically to the CEJ [65,66]. Polymerization shrinkage, the difference in the coefficient of thermal expansion among tooth substance and restorative material, and inadequate hybridization among collagen fibrils and the DBA caused by entrapped water at the interfibrillar spaces, can account for that [65,66].

Material selection for elevation and adhesive system employed for luting seem to be significant factors concerning the marginal adaptation of the restoration [29,39,41]. For DME, several materials have been used (microhybrid, nanohybrid, bulk-filled composites, siloranes, ormocers, self-adhesive resin cements, glass ionomers, resin-modified glass ionomers) at different viscosities (condensable, flowable, preheated) in one or more layers. However, researchers have no consensus regarding the material of choice for DME nor the effect of the technique on margin quality.

All studies that evaluated marginal integrity concluded that it was superior in enamel than in dentin [29,31,36,37,42,45,47]. Some authors support that, when bonding directly to dentin marginal adaptation is better [30,36,41,45], whereas others have demonstrated that DME does not negatively influence the quality of the restorative margins [2,31,32,33,34,46,49].

The incremental technique used in DME may positively influence marginal integrity; when using condensable composites, careful layering (3 layers) exhibits fewer gaps than no layering (1 layer) [30]. Indeed, Roggerdorf et al. [28] showed no difference in gap formation in dentin among DME groups and restorations cemented directly in dentin when using multiple layers of condensable resin. However, another study detected no difference between 1 layer and 2-layer groups [31]. Self-adhesive resin cements, also used for core build-up, should be avoided for DME since they manifest significantly more gaps than other materials after thermomechanical loading [28,29,30]. Some studies demonstrated the comparable performance of flowable and micro-hybrid composites when used for DME [34,36].

On the other hand, Scotti et al. [42] yielded that, at baseline, flowable composites provide adequate or even better marginal seal than nanohybrid and bulk-filled composites. However, they are more susceptible to degradation after thermomechanical loading and should be contraindicated [37,42]. Preheated composites are preferable [37].

In general, glass ionomers, resin-modified glass ionomers, resin-based composites, and bulk-filled composites are acceptable materials for DME since, so far, they do not seem to influence marginal quality [2,38,45]. When applying total-etch adhesives, the risk of over-etching dentin substrate in subgingival areas is substantial [19,41]. Juloski et al. [41] attribute the unsatisfying behavior of the specimens to this fact and subsequently to the type of the DBA used. Therefore, the authors strongly recommend the use of self-etch or universal adhesives for DME instead of total-etch ones.

### 3.3. Mechanical Performance

It seems that DME does not impact fatigue behavior [48], fracture resistance or failure pattern, or repairability regardless of the restoration material (ceramic/composite) [22,33], the elevation material [2,38,45], or the restoration design (inlays/onlays/endocrowns) [40,43,45,67]. When performing DME, the proximal extension of the restoration is limited and, therefore, the stress distribution is more favorable and so are failure patterns [33,68], even in higher loads and more eccentric forces [69]. Vertolli et al. [2] demonstrated that, when the restoration is cemented directly on enamel margins or the DME surface, it yields a significantly lower ceramic fracture rate (10%) than luting on the cementum margin (90%). According to the authors [2], great occluso-gingival proximal ceramic heights are associated with bulk fracture and, when they exceed 5 mm, DME needs to be considered.

When applying the multi-layering technique, the supplementary composite layer increases the bonding interfaces where failures commonly burst [44]. However, a recent finite element analysis [44] demonstrated that the maximum principal stress and the interfacial tensile stress between the DME layer and the other materials were below their failure strength. In contrast, the thickness of the DME layer did not affect them. Besides that, in DME, only a tiny portion of composite resin is used, which limits the polymerization stress in that area [30]. In a recent study [45] on endocrowns to rehabilitate endodontically treated premolars, DME increased their fracture resistance [40,45].

From the limited data concerning bond strength [13,70], there is no evidence that DME reduces the bond strength of the restoration to the proximal box regardless of the resin cement used (total etch/ self-etch) [13]. Failures mainly occurred at the dentin–composite rather than the composite–restoration interface [13].

### 3.4. Clinical Performance/Interaction with Periodontal Tissues

Bresser et al. [52] evaluated the clinical performance of 197 indirect restorations with DME in a 12-year time span. A 95.9% overall survival rate was identified, and among the eight failures, five of them referred to recurrent proximal decays. In another retrospective clinical study [54] with follow-up periods ranging from 6 to 21 years, no secondary caries was observed when DME was applied.

According to a clinical/histological study in humans [53], DME and subgingival restorations are compatible with periodontal health. Given that they are well-polished and refined [20,53], BW is not violated, and a strict supportive therapy along with good oral hygiene are followed [51,53,55]. Despite the low gingival index and plaque index rate, a high incidence of bleeding on probing is an anticipated result in the case of margins placed ≤2 mm from the bone crest (infringement of BW) [50]. This was further justified by a recent histomorphometric study reporting that the distance between composite restorations and bone crest should be at least 2 mm to avoid apical bone migration [71].

A recent systematic review [23] concluded that DME yields a better survival rate than surgical crown lengthening (SCL). Dablanca et al. [24] suggest DME when the lesion reaches the gingival sulcus till the junctional epithelium. When caries invades the connective tissue, an SCL needs to be performed. When it invades bone level and the tooth can be restored, a combination of SCL and DME is recommended; crown lengthening to the extent of the carious lesion would possibly expose the furcation. Therefore, it should be avoided [25].

However, the extent of BW violation may determine the biological reaction of hard and soft tissues [16]. For example, with a rigorous oral hygiene program, the infringement of a limited proximal area is better tolerated than a complete circumferential margin. Similarly, a randomized clinical study showed that subgingival proximal restorations impinging BW under a strict plaque control regimen yielded a similar plaque index, probing depth and bleeding on probing with SCL groups after six months [72]. These results signify that infringement of BW is not always equal to SCL.

The gingival attachment facing the deep lesion is destroyed [21]. DME does not lead to BW recreation but a healthy variable, comprised of a longer junctional epithelium alongside the material and a smaller connective attachment along the dentin underneath the composite [21].

## 4. Discussion

One of the main goals of current restorative dentistry is preserving healthy tooth structures. Therefore, minimally invasive preparation concepts and guidelines are preferred [59,73]. The rationale behind DME rests upon the coronal relocation of the restorative margin instead of displacing the margin of the periodontium according to the cavity limits.

The peculiar structure of dentin compared to enamel and the sensitivity of the DBA application procedure render dentin a very challenging substrate for bonding [60,74,75,76]. The presence of cementum also jeopardizes reliable adhesion [77]. Composite resins are subjected to shrinkage stress during polymerization, leading to debonding and subsequent interfacial gaps between the restorative material and the cavity walls. These gaps constitute active pathways for bacteria, fluids, ions, and molecules [37,41,42]. Restorations having margins in dentin and cementum are more prone to microleakage and thus postoperative sensitivity, marginal staining, and secondary caries emerge [78,79,80]. Secondary caries is the most common reason for restoration replacement and the primary etiologic failure factor [81,82,83,84]. Therefore, subgingival cavities that exceed CEJ require careful evaluation and handling; dry working place and precision during bonding procedures are prerequisites for an acceptable clinical outcome [85,86,87].

According to a clinical study, the operator and the treatment execution seem to be more determinant factors for clinical success than the material itself [88]. Therefore, any manner of facilitating clinical operations and reducing technique sensitivity should be seriously considered. Due to the lack of contamination simulation in laboratory (in vitro) studies, the benefits of DME may not be highlighted; compared to bonding one or more indirect restorations in deep subgingival margins under permanent contamination risk, placing a small increment of composite into the proximal box is easier. In addition, unlike conventional cements like glass ionomer in which excesses are eliminated after setting, resin-based ones need to be removed before polymerization, thus increasing the risk of bleeding in subgingival areas [8].

The most vulnerable part of an adhesive restoration is the dentin/adhesive interface [89]. With bonding procedures, we try to emulate dentinoenamel junction (51 Mpa), but the hybrid layer inevitably degrades gradually and so the 51 Mpa is the minimum microtensile bond strength that needs to be achieved with adhesive systems [90,91]. Therefore, IDS constitutes an integral step of indirect bonded restorations and should be incorporated in DME. It reduces bacterial microleakage and dentin hypersensitivity while enhancing bond strength [59]. Three-step total-etch and two-step self-etch systems are recommended for IDS since they yield superior durability, aging, and bond strength compared to single-step ones [92,93,94,95,96,97]. However, dentin over-etching in deep subgingival areas is a common phenomenon, so the use of total-etch systems should be avoided in DME [41]. A recent study by Carvalho et al. [98] examined the bond strength of five DBAs applied with three different methods (delayed dentin sealing, IDS, and IDS reinforced with low viscosity resin). They concluded that applying a flowable composite significantly improved the performance of unfilled/lightly filled adhesives. Therefore, three-step total-etch systems could be replaced by another DBA, avoiding the risk of over-etching having at the same time predictable outcomes concerning bond strength. It should be mentioned that the layer of flowable composite used to reinforce IDS is thin, it is applied not only inside the proximal box but on the whole exposed dentin surface (wherever IDS is performed), and it is independent of the elevation material in DME; it provides several advantages; among others, it prevents dentin re-exposure after conditioning, it interacts with the uncured resin of the acidic monomers from the oxygen inhibition layer improving polymerization of the DBA, it reduces adhesive permeability, and it improves coupling with resin cements [59].

DME relies on the transition zone among the composite applied in the first appointment and the resin luting agent in the cementation appointment, and so one could question the durability of the technique and its impact on restoration performance. It should be mentioned that the chemical bonding of free radicals is not the determinant factor for a resin-to-resin bond since they decline as the material ages and may be eliminated in 2.5 days [99]. Micromechanical interlocking and interpretating network matrices are more crucial instead; bond strength is determined by the depth of penetration of the resin cement monomers into the pre-existing composite [100,101]. Among other factors, increased polarity of the surface when in contact with water decreases the diffusion of monomers and thus methods that remove a few microns of the composite layer are preferred [99]. Gresnigt et al. [102,103] reported that the placement of a ceramic laminate veneer on a pre-existing aged composite does not affect its survival rate given that the composite is silica-coated and silanized before cementation. Similarly, we could assume that the existing composite used to elevate the subgingival margin does not affect the longevity of whichever indirect restoration cemented.

According to the literature, both glass ionomer and resin composite are well tolerated by periodontal tissues [104,105,106,107,108,109], and thus they could be used as elevation materials in DME. However, compared to composites, glass ionomers yield poor mechanical properties and insufficient long-term bond strength to the tooth surface. Therefore, the former constitute an appealing alternative for subgingival restorations if proper isolation is attainable [21]. It should be mentioned that the majority of the studies investigating the interaction of different materials with periodontal tissues concern cases of root coverage where filling, polishing and oral hygiene can easily be performed [21]. Rough surfaces favor the formation of dental plaque [110], and unlike the buccal sides of teeth, posterior proximal areas are not easily accessible and increase the operation difficulty for an optimal result.

Based on the consensus report by Jepsen et al. [111], the term “biological width” that refers to the apicocoronal dimension of gingival attachment alongside the root surface (junctional epithelium + supracrestal connective tissue) has been redefined to “supracrestal tissue attachment” (STA). There is no standard STA measurement, and it is the epithelial attachment that yields significant variability (1–9 mm), while connective tissue height is stable [112]. Therefore, in the case of a deep subgingival lesion, it is not possible for the clinician to define whether it remains within the epithelial attachment or whether it invades the connective tissue. The hemidesmosomal nature of the former renders it less resilient than the latter, which is comprised of horizontal collagen fibers firmly attached to the cementum. However, the epithelium has a superior adaptive capability; it is the only tissue that obtains attachment alongside the material [113,114]. Therefore, when it comes to subgingival restorations that compromise the integrity of STA, concerns should focus on the reaction of the connective component of STA instead of the epithelial one. Following this rationale, Ghezzi et al. [55] suggested a new classification system for deep proximal cavities based on rubber dam isolation capacity, regardless of the extent of the carious lesion. When a rubber dam can be placed, the working field is assumed to be limited within the epithelial area, and thus surgical intervention is not needed. On the other hand, in the case of connective tissue invasion, the field technically cannot be isolated and surgical procedures are required. Whether a rubber dam can be placed after creating a flap, ostectomy is not necessary. However, it is required when, after the flap opening, the depth of the caries does not permit proper isolation.

Forty-four studies were included in the present review. More than half (24) comprised in vitro studies, mainly referred to marginal adaptation (18 out of 24), fracture behavior (8 out of 24), and bond strength (2 out of 24) of the final restoration after DME application. Ten studies were reviews and case reports, while only six clinical studies were identified and taken into consideration. The absence of randomized controlled trials, which constitute the gold standard for establishing causal correlations in clinical research, is a limitation of this review. More well-designed clinical studies are needed to justify the efficiency of the described technique.

## 5. Conclusions

Within the limitations of this current study, the following conclusions may be made:

- DME is a promising technique that relocates the cervical margin coronally in a conservative way, thereby facilitating field isolation, impression taking, and cementation.
- It can be applied in both indirect and direct restorations.
- The available literature is limited mainly to in vitro studies. Therefore, randomized clinical trials with extended follow-up periods are necessary to clarify all aspects of the technique and ascertain its validity in clinical practice.
- For the time being, DME should be applied with caution respecting three criteria: capability of field isolation, the perfect seal of the cervical margin provided by the matrix, and no invasion of the connective compartment of BW.

## Figures and Tables

**Table 1:** Characteristics of the included studies.

Authors and Year of Publication	Type of Study	Tested Parameters	Study Design	Main Findings
Dietschi et al., 1998 [12]	Review	-	Presented new clinical concepts for adhesive cementation of composite and ceramic posterior restorations	A small portion of a composite resin can be placed over the existing subgingival margin, under rubber dam isolation and placement of a matrix.
Magne et al., 2012 [8]	Review	-	Presented technical details and clinical advantages of DME	DME is a noninvasive alternative for SCL and can be applied in both indirect and direct restorations.
Frese et al., 2014 [16]	Review/Case report	-	Presented technical details for DME in direct restorations.	BW violation determines periodontal tissues tolerance. Strict oral hygiene is required in subgingival restorations.
Dietschi et al., 2015 [17]	Review	-	Presented new clinical concepts for preparation and adhesive cementation of tooth-colored posterior restorations	DME facilitates field isolation, impression taking and adhesive cementation of indirect restorations with subgingival margins.
Kielbassa et al., 2015 [18]	Review/Case report	-	Reviewed the available literature concerning DME	DME facilitates operative procedures but is not clinically established yet.
Rocca et al., 2015 [19]	Review	-	Presented new clinical concepts for preparation and adhesive cementation of tooth-colored posterior restorations.	Modern preparation and luting concepts are influenced by tissue conservation principles.
Juloski et al., 2018 [20]	Review	-	Reviewed the available literature concerning DME.	DME is not clinically established yet.
Sarfati et al., 2018 [21]	Review/Case report	-	Reviewed the available literature concerning the effect of different materials used for subgingival restorations, on periodontium and presented three cases in which DME was performed instead of SCL.	DME seems well-tolerated by periodontal tissues.
Garaizabal et al., 2019 [22]	Systematic review	Fracture resistance	Evaluated fracture resistance and survival rate of inlays, onlays, and overlays fabricated by CAD/CAM ceramic, composite resin, resin nanoceramic and hybrid ceramic and investigated the effect of DME on fracture resistance.	DME did not affect fracture resistance of indirect restorations.
Mugri et al., 2021 [23]	Systematic review	Survival rate	Examined the survival rate of severely decayed teeth when restored using either SCL or DME.	Although there is a lack of high-quality trials examining surgical comparisons between the two techniques with long-term follow-up, DME has a better survival ratio than SCL.
Dablanca-Blanco et al., 2017 [24]	Case report	-	Examined seven clinical scenarios concerning deep proximal caries in molars.	If the carious lesion is limited to the epithelium level, DME can be performed. However, if it reaches the connective tissue or the bone crest, SCL is required.
Alhassan et al., 2019 [25]	Case report	-	Presented a case in which a combination of SCL and DME was performed.	When field isolation is possible, DME can be performed.
Butt, 2021 [26]	Case report	-	Presented technical details and clinical advantages of DME.	DME facilitates operative procedures but is not clinically established yet.
Elsayed, 2021 [27]	Case report	-	Presented technical details and clinical advantages of IDS, CDO and DME.	The combination of these techniques results in a minimally invasive restoration of extensive caries.
Roggendorf et al., 2012 [28]	In vitro	Marginal quality	Investigated the effect of DME on marginal quality of MOD composite inlays after TML, using one or three layers of different composites (forty MOD cavities/five groups: (1) DME with G-Cem, (2) DME with Maxcem, (3) DME in one layer of Clearfil Majesty Posterior, (4) DME in three layers of Clearfil Majesty Posterior, (5) without DME).	Three 1 mm layers of composite yielded superior marginal quality among the other groups. Self-adhesive resin cements as elevation materials are not indicated for DME.
Lefever et al., 2012 [29]	In vitro	Marginal adaptation	Evaluated the influence of DME on marginal adaptation of supragingival relocated margins of eighty-eight extracted molars using different elevation materials (Filtek Silorane, Clearfil AP-X, Clearfil Majesty Posterior, Clearfil Majesty Flow, RelyX Unicem, SDR, Vertise Flow) combined with different adhesive systems (Filtek Silorane Primer and Bond, Clearfil Protect Bond, Filtek Silorane Bond).	Marginal adaptation was material-dependent.
Frankenberger et al., 2012 [30]	In vitro	Marginal quality	Tested the DME effect on marginal quality of molar MOD glass ceramic inlays before and after TML, using one or three layers of different composites (Forty-eight MOD cavities/six groups: (1) DME with RelyX Unicem, (2) DME with G Cem, (3) DME with Maxcem Elite, (4) DME in one layer of Clearfil Majesty Posterior, (5) DME in three layers of Clearfil Majesty Posterior, (6) without DME).	Bonding directly to dentin yielded the fewest gaps. Marginal quality with three-layer DME was superior compared to one-layer. Self-adhesive resin cements as elevation materials are not indicated for DME.
Zaruba et al., 2012 [31]	In vitro	Marginal adaptation	Evaluated the impact of DME on marginal adaptation of molar MOD ceramic inlays after TML, using one or three layers of composite. (Forty MOD cavities/four groups: (1) margin in enamel, (2) DME in one layer of Tetric Composite, (3) DME in two layers of Tetric Composite, (4) without DME).	The composite–enamel interface showed the most gap-free margins. Marginal quality in DME was not significantly different from bonding directly to dentin.
Da Silva Goncalves et al., 2016 [15]	In vitro	Bond strength	Investigated the effect of DME (Adper Scotchbond 1XT, Filtek Z250) on μTBS of MO composite inlays to the dentin floor of the proximal box, luted with a conventional or a self-adhesive resin cement (twenty-five MO cavities/four groups: (1) without DME/luting with RelyX ARC, (2) DME in two layers of Filtek Z250/luting with RelyX ARC, (3) without DME/luting with G-Cem, (4) DME in two layers of Filtek Z250/luting with G-Cem).	DME increased bond strength in the proximal box with the self-adhesive resin cement.
Marchesi et al., 2014 [32]	In vitro	Marginal quality	Evaluated the influence of DME (Optibond FL, Filtek Supreme XTE flow) on marginal integrity of tenCAD/CAM lithium disilicate ceramic crowns before and after TML.	Marginal quality was not affected by DME.
Ilgenstein et al., 2015 [33]	In vitro	Marginal integrity/fracture behavior	Evaluated the impact of DME (2 layers of 1 mmTetric evo Ceram) on marginal integrity and fracture behavior of onlays after TML. (forty-eight MOD cavities/four groups: (1) without DME/feldspathic ceramic, (2) DME/feldspathic, (3) without DME/resin nanoceramic, (4) DME/resin nanoceramic).	DME did not affect fracture resistance. DME did not influence the marginal integrity of feldspathic onlays. Resin nano-ceramics were superior to feldspathic for both variables tested, especially in specimens without DME.
Spreafico et al., 2016 [34]	In vitro	Marginal quality	Evaluated the effect of DME on marginal quality of CAD/CAM crowns (pre-cured resin/lithium disilicate) before and after TML, using two layers of conventional or flowable composite (Forty preparations in molars/four groups: (1) DME with Filtek Supreme XTE/Lava Ultimate, (2) DME with Filtek Flow Supreme/IPS e.max, (3) DME with Filtek Supreme XTE/IPS e.max, (4) DME with Filtek Flow Supreme/Lava Ultimate).	DME did not influence marginal quality.
Müller et al., 2017 [35]	In vitro	Marginal quality	Evaluated the effect of DME on marginal quality of molar Cerec inlays luted with different materials (twenty-four MOD cavities, mesial boxes were elevated with Filtek Supreme/three groups: (1) luting with Scotchbond Universal + RelyX Ultimate, (2) luting with Monobond Plus, Syntac + Variolink II, (3) luting with Clearfil Ceramic Primer + Panavia SA Cement).	DME did not affect marginal integrity.
Köken et al., 2018 [36]	In vitro	Marginal sealing	Evaluated the effect of DME on marginal sealing of molar composite CAD/CAM overlays, using micro-hybrid composite or flowable composite. (thirty-nine MOD cavities/three groups: (1) DME with GC Essentia MD, (2) DME with GC Gaenial Universal Flo, (3) without DME).	Micro-hybrid and flowable composites are comparable in terms of marginal sealing ability. However, leakage scores were significantly lower when bonding directly to dentin.
Zavattini et al., 2018 [37]	In vitro	Microleakage	Investigated the influence of DME on microleakage of direct MOD composite restorations in thirty molars, using micro-hybrid (Premise dentin A3 Kerr), preheated micro-hybrid (Premise dentin A3 Kerr) or flowable composite (Premise flowable Kerr).	Flowable composite yielded the highest leakage scores.
Grubbs et al., 2019 [38]	In vitro	Marginal quality/fracture resistance	Examined the influence of DME on marginal quality and fracture resistance of CAD/CAM resin, nanoceramic onlays, using different materials (Seventy-five MOD cavities/five groups: (1) DME with Glass Ionomer Fuji IX, (2) DME with resin modified glass ionomer Fuji II LC, (3) DME with composite Filtek Supreme Ultra, (4) DME with Filtek bulk fill posterior restorative, (5) without DME).	All materials tested did not decline marginal quality nor fracture resistance of the restorations.
KöKen et al., 2019 [39]	In vitro	Microleakage	Evaluated the impact of DME and the adhesive system used on microleakage of MOD composite overlays (Twenty MOD cavities/two groups: (1) DME with G-aenial Universal Flo/luting with G-Cem Link Force + universal bonding agent GC G-Premio Bond, (2) DME with G-aenial Universal Flo/luting with G-Cem Link Force + three-step total-etch Kerr Optibond FL).	DME and adhesive system used for luting seems to affect microleakage.
Zhang et al., 2019 [40]	In vitro	Fracture resistance	Examined the influence of different restorative procedures on fracture resistance of RCT premolars. (Fifty MO cavities/five groups: (1) Unprepared teeth, (2) Endocrowns, (3) DME+ Endocrowns, (4) Crowns, (5) fiber posts+ crowns).	Endocrowns combined with DME yielded superior fracture resistance compared to other groups.
Juloski et al., 2020 [41]	In vitro	Marginal quality	Investigated the effect of DME on marginal quality of CAD/CAM overlays, using different materials. (Fourteen MOD cavities/two groups: (1) DME with total-etch adhesive Optibond FL + Premise Flowable in mesial margins, (2) DME universal adhesive Adhese universal + Tetric EvoFlow Bulk Fill in mesial margins).	Bonding directly to dentin provided better marginal quality. In DME, marginal quality is influenced by the materials used.
Scotti et al., 2020 [42]	In vitro	Interfacial gaps	Examined the impact of DME on marginal adaptation of direct composite restorations, using one or two layers of flowable resin or ormocer resin flow (forty-eight MOD cavities/six groups: (1) DME in one layer of Grandioso heavy flow + nanofilled composite Grandioso, (2) DME in one layer of Admira fusion Flow+ nanofilled ormocer Admira Fusion, (3) Like (1) in two layes, (4) Like (2) in two layers, (5) restoration with nanohybrid composite Filtek Supreme XTE without DME, (6) restoration with bulk nanofilled composite Filtek bulk-fill without DME).	Flowable resins are prone to interfacial degradation after loading.
Bresser et al., 2020 [43]	In vitro	Fracture strength	Evaluated the effect of DME (Optibond FL, Essentia Universal Composite) on fracture strength of lithium disilicate inlays and onlays. (Sixty cavities/four groups: (1) inlay without DME, (2) inlay with DME, (3) onlay without DME, (4) onlay with DME).	DME did not influence the fracture strength of the restorations tested.
Vertolli et al., 2020 [2]	In vitro	Structural/marginal integrity	Examined the influence of DME on structural and marginal integrity of CAD/CAM ceramic inlays, using glass ionomer (Fuji IX) or resin-modified glass ionomer (Fuji II LC). (Forty MOD cavities/four groups: (1) margin in enamel, (2) margin in cementum, (3) DME with Fuji IX, (4) DME with Fuji II LC).	DME led to decreased ceramic fracture rates. No difference was identified among glass ionomer and resin modified glass ionomer groups.
Chen et al., 2021 [44]	Finite element analysis (FEA)	Mechanical performance	Investigated the effect of design parameters of inlays on DME.	DME did not influence fracture resistance of inlays.
Zhang et al., 2021 [45]	In vitro	Fracture resistance/microleakage	Tested the impact of DME on fracture resistance and microleakage of RCT premolars restored with ceramic endocrowns, using a bulk-fill (bulk-fill Smart Dentin Replacement) or a conventional composite (Z350 XT). (Eighty MO cavities/four groups: (1) margin in enamel, (2) DME with bulk-fill composite, (3) DME with conventional composite, (4) without DME).	DME increased fracture resistance of premolar endocrowns but not microleakage.
Alahmari et al., 2021 [46]	In vitro	Marginal adaptation	Evaluated the effect of DME on marginal adaptation of CAD/CAM lithium disilicate crowns. (Forty preparations/four groups: (1) margins in enamel, (2) DME with flowable composite, (3) DME with composite resin fillings, (4) DME with composite resin fillings).	The implementation of DME had a good effect on marginal integrity of the cervical margins.
Da Silva et al., 2021 [47]	In vitro	Marginal sealing	Studied the influence of gingival margin position (1 mm above or below CEJ or DME) and the adhesive strategy used (Enamel + etch-and-rinse adhesive (ERA) Adper Scotchbond 1XT (SB1XT)/Dentin + SB1XT/DME + SB1XT/Enamel + self-etching adhesive (SEA) with enamel selective etching Clearfil SE Bond (CSE)/Dentin + CSE/DME + CSE) on marginal sealing of twelve MOD composite inalys (Gradia Indirect).	A perfect sealing ability was evidenced for groups with enamel margins. When CSE adhesive was applied similar nanoleakage values were achieved regardless the gingival margin position.
Grassi et al., 2021 [48]	In vitro	Fatigue behavior, stress distribution	Evaluated the effect of DME and restorative materials (leucite-reinforced glass-ceramics/indirect resin composite) on the fatigue behavior and stress distribution of fifty-two maxillary molars restored with MOD inlays.	DME was not negative for fatigue and biomechanical behaviors. Resin composite inlays were more resistant to the fatigue test, although the failure mode was more aggressive.
Moon et al., 2021 [49]	In vitro	Interfacial gaps	Evaluated the effect of DME (resin modified glass ionomer) on interfacial gap formation of twelve CAD/CAM lithium disilicate inlay margins before and after TML.	DME with resin modified glass ionomer reduced the extent of interfacial gap formation before and after the aging simulation.
Ferrari et al., 2017 [50]	Clinical	Periodontal health	Tested the effect of DME (GPremio Bond, Flow resin GC Co) on periodontal health of thirty-five lithium disilicate crowns at baseline and after 12 months.	A higher incidence of BoP is anticipated in case of BW violation.
Bertoldi et al., 2018 [51]	Clinical	Inflammatory response	Investigated the effect of DME on inflammation response of periodontal tissues surrounding eight endodontically treated teeth restored with post-and-core restorations.	There was no statistically significant difference in inflammation degree after DME.
Bresser et al., 2019 [52]	Clinical	Clinical performance	Investigated the impact of DME on clinical performance (secondary caries, root caries, fracture, debonding, severe periodontal breakdown, pulpal necrosis) of 197 indirect restorations after 12 years of function.	DME did not influence the survival rate of the indirect restorations tested (95.9%).
Bertoldi et al., 2019 [53]	Clinical/histological	Inflammatory response	Evaluated the effect of DME on the clinical and histological reaction of periodontal tissues surrounding twenty-nine posterior teeth.	DME is well-tolerated by periodontal tissues given that BW is not violated and a strict supportive therapy is followed.
Dietschi et al., 2019 [54]	Clinical	Clinical performance	Examined clinical performance of twenty-five indirect adhesive restorations in which IDS, CDO, and DME were performed.	IDS, CDO, and DME favor the survival of indirect restorations.
Ghezzi et al., 2019 [55]	Clinical	Inflammatory response	Investigated the effect of three different approaches for rehabilitation of deep lesions (non-surgical DME, Surgical DME- gingival approach, surgical DME- osseous approach) on inflammatory response of periodontal tissues in fifteen cases.	If the connective compartment of BW is not infringed, DME is compatible with periodontal health.

Abbreviations: deep margin elevation (DME), surgical crown lengthening (SCL), mesial-occlusal-distal (MOD), thermomechanical loading (TML), biological width (BW), mesial-occlusal (MO), bleeding on probing (BoP), immediate dentin sealing (IDS), cavity design optimization (CDO), root canal treated (RCT).

## Data Availability

Not applicable.

## Abbreviations

DME	Deep margin elevation
CEJ	Cementoenamel junction
IDS	Immediate dentin sealing
SCL	Surgical crown lengthening
MOD	Mesial-occlusal-distal
TML	Thermomechanical loading
BW	Biological Width
MO	Mesial-occlusal
BoP	Bleeding on probing
CDO	Cavity design optimization
RCT	Root canal treated
DBA	Dentin bonding agent
OIL	Oxygen inhibition layer
STA	Supracrestal tissue attachment
